# Biologically Active Compounds in Mustard Seeds: A Toxicological Perspective

**DOI:** 10.3390/foods10092089

**Published:** 2021-09-03

**Authors:** Julika Lietzow

**Affiliations:** Department of Food Safety, German Federal Institute for Risk Assessment, Max-Dohrn-Straße 8-10, 10589 Berlin, Germany; julika.lietzow@bfr.bund.de

**Keywords:** mustard, *Sinapis alba*, *Brassica nigra*, *Brassica juncea*, glucosinolates, isothiocyanates, bisphenol F, erucic acid, allergy, toxicology

## Abstract

Mustard plants have been widely cultivated and used as spice, medicine and as source of edible oils. Currently, the use of the seeds of the mustard species *Sinapis alba* (white mustard or yellow mustard), *Brassica juncea* (brown mustard) and *Brassica nigra* (black mustard) in the food and beverage industry is immensely growing due to their nutritional and functional properties. The seeds serve as a source for a wide range of biologically active components including isothiocyanates that are responsible for the specific flavor of mustard, and tend to reveal conflicting results regarding possible health effects. Other potentially undesirable or toxic compounds, such as bisphenol F, erucic acid or allergens, may also occur in the seeds and in mustard products intended for human consumption. The aim of this article is to provide comprehensive information about potentially harmful compounds in mustard seeds and to evaluate potential health risks as an increasing use of mustard seeds is expected in the upcoming years.

## 1. Introduction

Mustard has been known as one of the oldest condiments ever and has been considered as one of the most widely grown and multifunctional plant in the world for thousands of years. The first known cultivars and use of mustard plants dating back to 3000 B.C. [1]. The following section gives a short overview about mustard plants, their taxonomic classification and the economic relevance of mustard seeds.

Mustard plants belong to the commonly known mustard family Brassicaceae of the order Brassicales (previously denoted as Capparales) including over 330 genera and over 3700 species that are distributed worldwide [2]. Notable characteristics of this family are the four sepals in median position of the flowers followed by four alternating petals that are arranged in a crossform referring to the old family name Cruciferae. The presence of organosulphur compounds is also a unique characteristic of this plant family.

**Figure 1:**
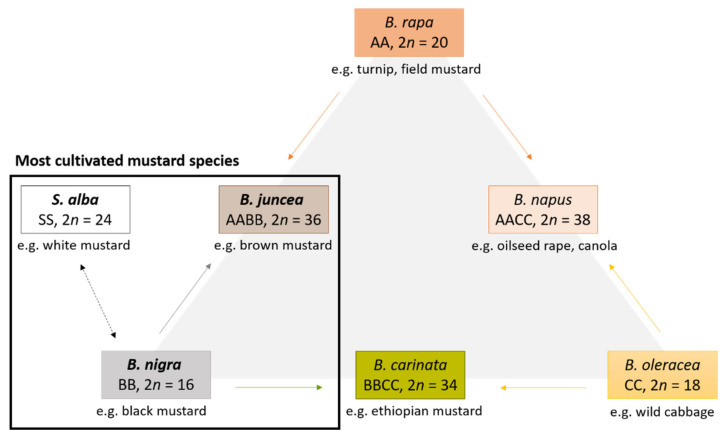
Taxa, genomes, chromosome number (n) and genetic relationship among Brassica species represented by U’s triangle. The most cultivated mustard species that are subject of this article are framed in black.

The most cultivated varieties within this family almost all belong to the six species *Brassica rapa*, *Brassica juncea*, *Brassica nigra*, *Brassica carinata*, *Brassica oleracea* and *Brassica napus* in which also two of three of the most important mustard species are included (*B. juncea* and *B. nigra*). Based on cytogenetic and hybridization experiments of the Korean botanist U Nagaharu (1935), a well-known model named “U’s triangle” have demonstrated the genetic relationship among these different species. The triangle is based on the theory that the genomes of three ancestral diploid species of Brassica combined to give rise to three common tetraploid vegetables and oilseed crop species. Figure 1 illustrates the triangle and shows the main forms *B. rapa*, *B. nigra* and *B. oleracea* with their own elementary genome A, B and C. The three amphidiploid species *B. juncea* (AB), *B. napus* (AC) and *B. carinata* (BC) are natural hybrids of the diploids. A genetic relationship exists also between *B. nigra* and the genera *Sinapis* L. in which the species *Sinapis alba,* belonging to one of the important mustard species*,* is included [1,3,4,5].

Amongst the approximately 40 species of mustard plants in the Brassicaceae family, three types, derived from the genera *Sinapis* and *Brassica*, are mainly commercially cultivated throughout the world and used to make the mustard condiment.

The genus *Sinapis* L. contains several different types of mustard species, including *Sinapis alba* (L.) (white mustard, syn. *B. hirta* Moench or *B. alba* Linn) or *Sinapis arvensis* (charlock mustard). As mentioned above the cultivated form *S. alba* L. is phylogenetically close to Brassica species and occasionally referred to as *Brassica alba* or *B. hirta.* The seeds of *S. alba* are light straw yellow-colored and are slightly larger than the seeds of *B. nigra* and *juncea*. White mustard), believed to be native to the Mediterranean region, is the most used mustard species in Europe [1,6]. In warm regions, the mustard plant is primarily cultivated for its seeds, as a spice and for its high oil content, whilst in cold regions it is grown as catch crop, green fodder crop or as green manure. Varieties of mustard blends made from white mustard are available on the market. For example the American ballpark-style mustard is made from the white seeds, blended with vinegar and spices and usually coloured with turmeric [7]. Characteristic of the white mustard seeds is the glucosinolate sinalbin, which is enzymatically hydrolysed to 4-hydroxybenzyl isothiocyanate contributing significantly to the irritating odour and the sharp flavour of the mustard. However, white mustard is milder in taste than other mustard species. The seeds are often used for pickling gherkins or mixed pickles or making sausages [7]. Several varieties of yellow mustard exist with different qualities such as earlier maturing, nematode-resistance or altered content of glucosinolates or erucic acid. According to the catalogue of varieties of agricultural plant species more than 100 varieties of *S. alba* L. are listed [8].

*Brassica nigra* (L.) W.D.J. Koch (black mustard, syn.: *S. nigra, B. sinapioides*) is widely cultivated for its blackish brown-red seeds which are slightly bitter and more pungent than the seeds of the white (*S. alba)* or brown (*B. juncea*) mustard. Sinigrin is the major glucosinolate in the seeds of black mustard and can be hydrolysed to allyl-isothiocyanate (AITC) giving the characteristic of a pungent irritating odour [9]. Black mustard is commonly used as spice, potherb and as source of oil, however in the Unites States (U.S.) and Europe difficulties in harvesting reduce its popularity [7]. Likewise, the plant plays an important role in traditional medicine since eternal times, either used internally or externally. Black mustard oil is also utilized for production of soap and for medicinal remedies [10]. When black mustard seeds are grounded to meal and mixed with vinegar, a pungent condiment occurs. Among the different mustard varieties, the traditional Dijon mustard originated in the city of Dijon in France, is very popular and made from black or brown mustard seeds blended with wine (vinegar) and/or verjuice and seasonings. If vinegar is added to a ground mixture of seeds of black and white mustard a milder blend is produced (e.g., German mustard) [7].

Plants of *Brassica juncea* (L.) Czern. and Coss. (brown mustard, syn. *B. integrifolia*) are grown worldwide, in North America and Europe mainly for the seeds to be used as condiment, in the Indian subcontinent for oil production, and in Asiatic countries like China and Japan the mustard plant is used as a root and leaf vegetable [1,6]. *B. juncea* plants are shorter and have larger seeds than its relative *B. nigra. B. juncea* is more suitable for its agricultural cultivation and mechanical harvesting than *B. nigra* due to its higher growth and its pods, which are less prone to bursting. Therefore, brown mustard enables less labour-intensive and cost-effective production and increasingly replaces cultivation of black mustard [9]. *B. juncea* is a hybrid form derived from interspecific crosses between *B. nigra* and *B. rapa* giving it the characteristics of rapid growth from *B. rapa* and the mustard oil of *B. nigra*. Several subspecies of *B. juncea* exist with different morphologies and characteristics. Besides the oilseed types, vegetable and root type forms are cultivated for its edible leaves, inflorescences, stems and roots [1,11]. Due to its close relative to *B. napus* (oilseed rape, canola) a canola variety known as *Brassica juncea var. juncea canola* was developed from *B. juncea* resulting in low erucic acid and glucosinolate content and comparable oil and meal quality as canola (rapeseed) species [12,13]. The most important constituent in brown mustard is the natural glucosinolate sinigrin which on hydrolysis yields up to 1.4% allyl-isothiocyanate, known as volatile oil responsible for the pungent taste of brown mustard. *B. juncea* is also used as fodder and in traditional medicine, e.g., as diuretic or stimulant [10].

The world’s largest producer of mustard seeds in the last years was Nepal, accounting for more than 32% of the global production in 2019, followed by Russia with 25% and Canada with 21%. In the recent past, the largest importers of mustard seeds across the globe were the U.S. and Germany, followed by France, however the import values strongly vary depending on the different mustard species intended to be market. In this case, North America is the major market for yellow mustard used for the condiment industry, whereas Europe is important for brown mustard for use as a condiment, often in the form of specialty mustards. The Asia–Pacific region is the major market for brown mustard used as spicy cooking oil and condiments [14]. According to data provided by FAOSTAT (Food and Agriculture Organization of the United Nations), the countries UK (221 K tonnes), Germany (202 K tonnes) and Italy (190 K tonnes) had the highest volumes of prepared mustard consumption in 2019, together comprising 44% of total consumption [15]. In 2019, the average consumption of mustard per capita in Germany was around 900 g [16]. So far, the average daily consumption of mustard might be in the range from 1 to 2 g and it is very common to consume around 10–20 g mustard (as condiment) during a meal.

Mustard plants, especially the seeds, are used as or in food in many different forms and due to different functional properties. Processed foods are often mixed with natural mustard seeds, such as in pickled gherkins or small white onions. Mustard is also used as an ingredient in many ready-cooked dishes like crackers, appetizers, various flours and dehydrated products for soups. Moreover, various spicy sauces, vinaigrettes and mayonnaises often contain mustard condiment. Mustard may even be present in baby food. The risk of contamination is high, e.g., in fast food restaurants or snack stands where several products with mustard are handled [9,10].

To put it briefly, the seeds of mustard plants are characterized by various nutritional and functional properties and the use in the food and beverage industry is constantly growing. As mustard plants contain several bioactive compounds, the purpose of this review is to present a comprehensive overview about potentially harmful substances in the seeds from a toxicological perspective and to evaluate possible risks to human health.

## 2. Biologically Active Compounds in Mustard Seeds

Long ago, mustard was considered as medicinal plant rather than as culinary one pointing out the existence of biologically active compounds in the plants and their seeds. In general, these so-called “bioactive compounds” are typically present in various natural-based sources characterized by specific functionalities. In recent years, the research interest in these phytochemicals increased exponentially in various commercial industries. Moreover, scientific and (bio)technologic developments have led to immense nutritional discoveries and product developments resulting in increasing numbers of food products with potential medical and health benefits. Due to its broad use as nutraceutical or functional constituents for several health purposes, biologically active compounds have entered the market in many different forms (e.g., as food supplements), partially without reliable scientific basis with respect to efficacy or safety [17,18].

The growing consumer interest towards different taste preferences and healthy eating habits increases the use of mustard seeds and products made thereof. Due to its different functional and technological properties, mustard is gradually utilized in a wide-range by the food and beverage industry as well as in the cosmetic and pharmaceutical industry Moreover, the use of mustard plants has gained increasing interest for several non-food uses. For instance, seed meal of yellow mustard (*S. alba*) was shown to be efficient in controlling weeds, and oriental mustard (*B. juncea*) seed meal has been used as a broad-spectrum pesticide to control nematodes, insects, and fungi [19,20]. Current and future studies will undoubtedly focus on the health benefits of mustard plants and seeds whereas information on undesirable and antinutritional compounds in these products intended for human consumption will tend to fade into the background. Therefore, this article underscores and evaluates those compounds in mustard seeds potentially exerting undesired effects in humans.

### 2.1. Glucosinolates

The unique properties of glucosinolates and their breakdown products isothiocyanates, so called mustard oils, providing the typical flavour and (bitter) taste of mustard plants. Especially their seeds have been known for hundreds of years [21]. Since then, up to 200 different glucosinolates have been reported, predominantly in the plant families of the order Brassicales, but also in plants of the genus Drypetes [22,23].

Glucosinolates may be defined as amino acid-derived sulphur-containing glycosides sharing a generic chemical structure consisting of thiohydroximates carrying an S-linked b-glucopyranosyl moiety and an O-linked sulfate residue, and with a variable side chain (R). Until now, more than 200 side-groups (alkyl, alkenyl, aryl, indolyl) have been identified. Occasionally, further substituents are attached to O, S or N atoms of the side chain or glucosyl moiety [22,23,24].

Glucosinolates can be divided into three chemical groups including aliphatic, indole and aromatic glucosinolates depending on the amino acid precursor. Aliphatic glucosinolates are derived from alanine, leucine, isoleucine, valine and methionine. Indolic group are derived from tryptophan and aromatic glucosinolates are derived from phenylalanine and tyrosine, respectively. The majority of those are the aliphatic glucosinolates derived from chain-elongated methionine [25,26,27].

**Figure 2:**
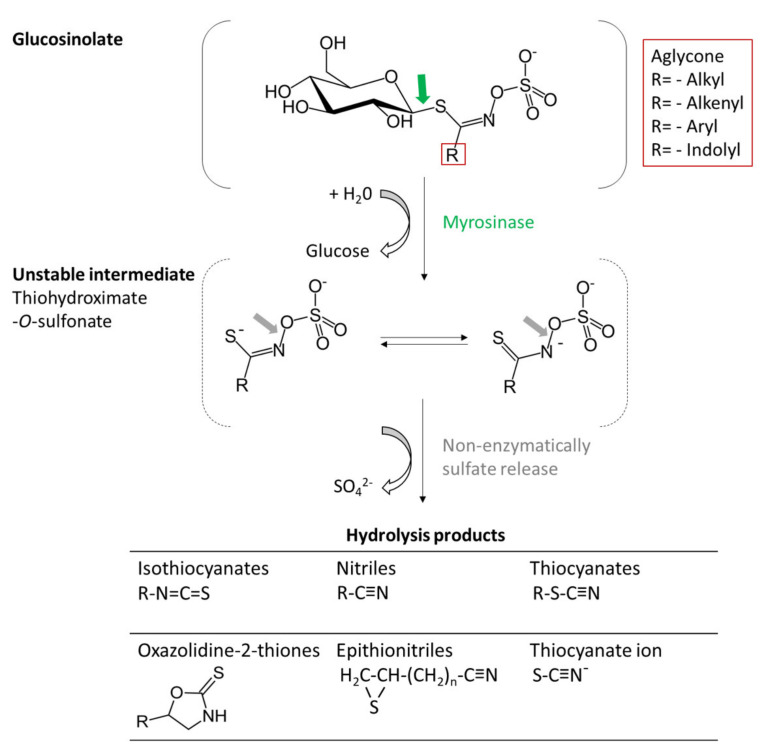
Enzymatic processing of glucosinolates by myrosinase into hydrolysis products.

Most of the hydrophilic glucosinolates are chemically and thermically stable. However, they can be converted into diverse breakdown products by specific ß-thioglucosidases, referred to as myrosinases, which are present in glucosinolate-producing plants but also in fungi and in bacteria commonly associated with gut microflora [28,29] (Figure 2).

In that regard it is of interest, that significant amounts of metabolites of glucosinolates (largely dithiocarbamates) were excreted via the urine of healthy human volunteers after eating brassica vegetables, even when myrosinase has been completely heat-inactivated. The excretion falls to negligible levels when the microbiota was disturbed, e.g., due to antibiotic treatment [30]. Therefore, bacterial myrosinase activity can cause the breakdown of glucosinolates in the distal gut, leading to release of several glucosinolate metabolites, e.g., isothiocyanates into the faecal stream and/or the urine.

In intact plants, myrosinases are separated from its substrates, e.g., in different cells or in different intracellular compartments. Damage of the plant tissue in case of preparing or chewing results in enzyme release. In the presence of water myrosinase initiates upon catalyzation by ascorbic acid hydrolytic cleavage of the β-glucosyl moity forming glucose, hydrogen ion and an unstable aglycone (thiohydroxamate-O-sulfonate). It is of note, that myrosinases are activated to various degrees by ascorbic acid, and in some instances the enzyme is almost inactive in its absence [22]. After the non-enzymatically release of the sulfate residue various volatile and non-volatile compounds derive, which exert different biological activities such as plant defence against insects or phytopathogens, or as attractants, and also give the specific flavour and pungency of mustard [31,32].

The hydrolysis products include isothiocyanates, previously known as “thioglucosides” or “mustard oils”, thiocyanates, nitriles, epithionitriles, oxazolidine-2-thiones (5-vinyl-2-oxazolidinethione and 5-vinyl-1,3 oxazolidine-2-thione), and indol derivatives depending on the aglycone structure and reaction conditions (e.g., pH value, ferrous ion concentration, specifier proteins) (Figure 2). Considering plant biology, these alternative breakdown products provide an additional level of plant defence [33,34].

Under physiological conditions, isothiocyanates contribute to 60–90% of the total glucosinolate breakdown products. After release of glucose, the thiohydroximate-O-sulfonate is formed, which can be spontaneously degraded by a Lossen-like rearrangement to the relatively stable isothiocyanates.

Isothiocyanates are very reactive and toxic for microbes, nematodes, fungi and insects, however many species of the Brassicaceae are able to promote alternative activation pathways by the action of specifier proteins. In the presence of these proteins, formation of isothiocyanates is reduced in favour of alternative breakdown products [35].

For instance, the presence of nitrile specifier proteins (NSPs) leads to an increased formation of nitriles [36,37], whereas, the occurrence and activity of the epithiospecifier protein (ESP) leads to the generation of epithionitriles from alkenyl-glucosinolate aglycons as well as nitriles from non-alkenyl-glucosinolate-aglycons. Moreover, it was shown that at physiological pH, isothiocyanates are the major products, whereas nitriles are formed at more acid pH [38].

Each type of Brassica vegetables including mustard plants show a characteristic glucosinolate composition and most species contain a limited number of glucosinolates [22]. The amount and composition vary among the different plant organs such as roots, leaves, stems and seeds, mainly with the highest concentrations often found in the reproductive tissues (florets, flowers and seeds). Therefore, for quantification of glucosinolates and their corresponding breakdown products, the seeds seem to be the best bulk source. The glucosinolate profile also depends on various environmental and ecophysiological factors such as plant nutrition, water availability, plant age and plant cycle [39].

Table 1 and Table 2 give an overview of glucosinolates and their corresponding desulfated breakdown products identified in the mainly cultivated mustard species *S. alba*, *B. nigra*, *B. juncea* [22,24,29,40]. The mustard species *S. alba, B. nigra* and *B. juncea* show large ranges between the highest and lowest levels of total glucosinolate content as well as different content of predominant glucosinolates, rise up to 200 µmol/g seed.

**Figure 3:**
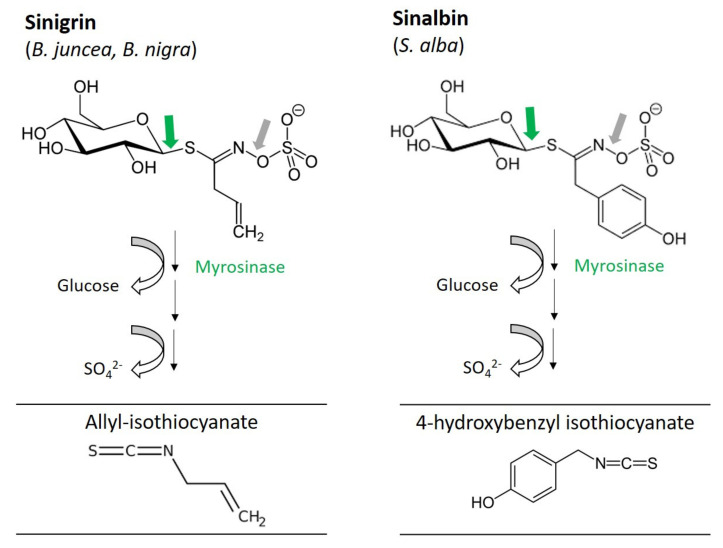
Main glucosinolates and corresponding hydrolysis products in brown (*B. juncea*), black (*B. nigra*) and white (*S. alba*) mustard seeds.

Sinalbin and sinigrin whose chemical structure was elucidated by Ettlinger and Lundeen [48,49] belong to the main glucosinolates in mustard seeds. In brown mustard (*B. juncea*) and black mustard (*B. nigra*) seeds sinigrin is the predominant compound degrading to allyl-isothiocyanate (AITC) after hydrolysis, whereas, the main glucosinolate of white mustard (*S. alba*) is sinalbin yielding 4-hydroxybenzyl isothiocyanate (29) (Figure 3).

The concentration of glucosinolates and their breakdown products in mustard plants, especially in the seeds, is largely dependent on the activity of myrosinase or related bacterial enzymes or further chemical transformation prior to consumption [26].

In case of mustard as food one has to distinguish between the intake of whole not-processed seeds as seasoning in several dishes, and processed seeds, for example as mustard flour made from ground, peeled and non-degreased seeds or as oil made by pressing the seeds. Prepared mustard is mostly composed of a mixture of ground mustard seed and/or mustard flour and/or mustard cake [9,10].

In general, glucosinolates are relatively stable in the seeds until cell damage results in chemical degradation involving myrosinase-catalysed hydrolysis. Thermal treatment leads to inactivation of myrosinase via denaturation [50] and preserve most of the glucosinolates. Van Eylen et al. showed that the optimum temperature of myrosinase activity of mustard seeds from *S. alba* was at 60 °C, however the activity decreased when the temperature of the experimental system was higher and was influenced by several other factors such as pH or presence of ascorbic acid [51].

This occurs for example when whole mustard seeds are added as spice to dishes which are cooked, like soups or stews. In this case, intact glucosinolates might reach the colon, however a certain amount of the glucosinolates seem to be hydrolysed in the stomach or can be absorbed via passive transport or via diffusion in the small intestine. In the colon, several bacteria strains are able to hydrolyse glucosinolates to form isothiocyanates, amines or nitriles depending on the type of bacterial myrosinase-like activity. Usually, cooking reduces the concentration of glucosinolates, partly through thermal breakdown and partly to leaching of the intact glucosinolates and their derivatives into the cooking liquid. Besides, heat treatment inhibits the activity of myrosinase through denaturation of the enzyme [52,53]. Therefore, the processing method can make a very large difference, both to the intake of glucosinolates, and to the bioavailability of their breakdown products.

The pattern of intact glucosinolates and breakdown products upon heat treatment is influenced by the thermal stability of the corresponding molecules. For instance, indol glucosinolates—one of the best-known representatives is progoitrin—seem to be less stable than aliphatic ones such as sinigrin, which is the predominant glucosinolate in black and brown mustard seeds. However, sinigrin totally disappears upon boiling, while 5-vinyloxazolidine-2-thione (derived from progoitrin) and 3-methyl-sulphinylpropylisothiocyanate (derived from glucoiberin) may partly escape decomposition [54].

Comparison between different mustard seed preparations showed that the seed alone had higher sinigrin and lower content of the breakdown product isothiocyanate compared to mustard preparations, whereas the wholegrain-style mustard contained lower sinigrin and higher isothiocyanate levels. This suggests that during mustard preparation, the enzyme myrosinase can break down sinigrin in the presence of high salinity and acidity from added ingredients, such as salt or vinegar [55]. Isothiocyanates are volatile and water-soluble. It can be expected that they largely disappear after processing steps using high temperatures and soaking in water, but those processes are commonly not included during mustard preparation.

Interestingly, in a thesis, it was shown that beside allyl-isothiocyanate derived from sinigrin, sulforaphane derived from glucoraphanin also corresponds to the most abundant breakdown product detected in different samples of table mustard. In this case, concentration of allyl-isothiocyanates ranged from 4 to above 200 mg/100 g fresh weight whereas sulforaphane ranges from 24 to above 188 mg/kg. These unexpected results of sulforaphane quantification may be explained by the fact that table mustard is a mixture and probably also contain glucoraphanin-containing ingredients [56].

Consumption of mustard seeds, either whole or after preparation, may lead to the intake of intact glucosinolates as well as their different breakdown products. One has to note, that the pungent taste of different prepared mustard samples strongly depends on levels of allyl-isothiocyanates [57]. The dietary intake of glucosinolates in general have been reported ranging from 2 to 29 mg/day depending on population groups and countries [58]. However, dietary intake of glucosinolates through the consumption of mustard as well as exposure data on different types of glucosinolates, especially of sinigrin and sinalbin, and their corresponding breakdown products such as allyl-isothiocyanates, are not available.

#### 2.1.1. Toxicological and Antinutritional Effects

Many plants of the Brassicaceae family containing glucosinolates are used for human and animal nutrition. Glucosinolates themselves are biologically inactive, however various breakdown products exert a variety of toxicological and antinutritional effects in animals whereas the information on adverse effects in humans is limited.

Numerous studies confirm the negative effects that occur in animals after feeding of glucosinolate-containing rapeseed cake (de-oiled seeds). After oil extraction from oilseed crops, such as rapeseed (*B. napus)* or white mustard (*S. alba)*, the hydrophilic glucosinolates remain in the seed meal fraction (by-product) used in animal diets [25]. The presence of the glucosinolates sinigrin and progoitrin in the diet is strongly associated with bitter taste and is responsible for reduced palatability corresponding to a lower intake of glucosinolate containing animal diets [26,59].

Although progoitrin is a non-bitter glucosinolate, it produces more profound bitter taste compared to sinigrin due its degradation to the extremely bitter compound goitrin by myrosinase or by heat treatment [60]. In Annex I of Directive 2002/32/EC, vinyl thiooxazolidone (5-vinyloxazolidine-2-thione (5-VOT), known as goitrin) and volatile mustard oil expressed as allyl-isothiocyanate are listed as undesirable substances with maximal amounts for animal feed. Humans are also sensitive to the strong flavours of glucosinolate breakdown products, and these compounds are therefore important determinants of intentionally or unintentionally flavour [61].

The main toxic effects in animals can be described as decreased feed intake, growth depression, enlargement of liver and kidneys, structural and functional alteration of the thyroid gland, and reprotoxic effects such as embryo mortality in mammals and reduced egg production in birds, amongst which the adverse effects on thyroid metabolism are the most thoroughly studied. Even though the extent of the detrimental effects both on productivity and on health vary within the animal species and the amount of glucosinolates, it is suggested that the formation of isothiocyanates, thiocyanates, oxazolidinethiones and nitriles from glucosinolates strongly contribute to the observed toxicological effects [62,63].

#### 2.1.2. Goitrogenic Effects

The reduction of the livestock feeding quality of seed meal following oil extraction is largely due to the presence of thiocyanate ion (SCN-), isothiocyanates and oxazolidine-2-thiones, which all have been shown to be goitrogenic [64].

At neutral pH, myrosinase-initiated hydrolysis of glucosinolates mainly yield isothiocyanates which are relatively stable in aqueous solutions. In comparison and especially at higher pH, isothiocyanates from glucobrassicin and neoglucobrassicin, which belong to indolylglucosinolates, as well as sinalbin may degrade to free thiocyanate ion and further metabolites [65]. McGregor et al. reported in oil-free seed meal from four cultivars of *B. juncea* thiocyanate ion concentration of 5.4 to 6.9 µmol/g whereas the amount of volatile isothiocyanates correspond to 116.9 to 145.9 µmol/g [47]. Mustard seeds of *S. alba* contain the glucosinolate sinalbin, which under physiological condition (pH 5–7) is decomposed mainly to 4-hydroxybenzylalcohol and thiocyanate ion. Under acidic conditions other degradation compounds were identified, such as 4-hydroxybenzyl cyanide or 4-hydroxybenzylnitrile (2-(4-hydroxyphenyl)acetonitrile) [66]. Paunovic et al. investigated sinalbin degradation products in ground yellow mustard seeds and paste. They identified 2-(4-hydroxyphenyl)acetonitrile as the major degradation product in ground seeds, whereas the most abundant sinalbin degradation product in mustard paste was 4-(hydroxymethyl)phenol. Other degradation compounds identified were 4-methyl phenol, 4-ethyl phenol, 4-(2-hydroxyethyl)phenol and 2-(4-hydroxyphenyl) ethanoic acid [67].

The thiocyanate ion formed by degradation of glucosinolates is known to be a competitive inhibitor of the sodium/iodide (NIS) symporter located on the basolateral membrane of the thyroid follicular cell. Consequently, the iodide uptake by the thyroid gland is impaired which can lead to reduced synthesis of thyroid hormones [68]. The effect is only evident in iodine-deficient diets and increased iodine intake can prevent the adverse effects.

This is not the case for goitrin. Isothiocyanates that are derived from progoitrin (2-hydroxy-3-butenyl glucosinolate) cyclize to produce 5-vinyloxazolidine-2-thione, known as goitrin. Goitrin is a potent inhibitor of the thyroid peroxidase, which plays a central role in the thyroid hormone biosynthesis by regulating organification, iodination and coupling reactions to form thyroid hormones (T3 and T4) in the thyroid gland. This leads to suppressed thyroxine secretion and reduced serum tetraiodothyronine (T4) concentration and can result in a compensatory increase in thyroid glandular mass (known as goiter- hyperplasia and hypertrophy). This has been shown in several animal species. Adverse effects in animals were observed with different concentrations of progoitrin/goitrin in the diet, ranging from 71 to 755 µmol/100 g depending on species investigated. In general, ruminants seem to be less sensitive to dietary glucosinolates, unlike pigs, which are severely affected by dietary glucosinolates compared to rabbit, poultry, and fish [63,69].

Besides isothiocyanates, nitriles have been reported to exert goitrogenic effects [70] however only sparse descriptions exist about the mode of action of nitriles on the thyroid gland. More reliable data report adverse effects on liver and kidney functions of this substance class [62]. The formation of nitriles from glucosinolates is favoured under acid conditions or in the presence of Fe(II) ions, whereas epithiospecific protein (ESP) promotes the formation of epithionitriles.

It was shown that nitriles were the dominant glucosinolate products from hydrolysis of aliphatic glucosinolates in cabbage presumably due to the effect of endogenous Fe(II) ions [71]. However, limited data exist regarding the amount or formation of nitriles consuming mustard seeds. Cole et al. showed negligible concentrations of nitriles in seeds of *B. juncea* or *B. nigra* [72] and Choi et al. detected only very small amounts of organic nitriles in serum of rats treated with sinigrin, the predominant glucosinolate in mustard seeds [73].

In principle, exposure of isothiocyanates and goitrin derived from glucosinolates, predominantly sinigrin and gluconapin occurring in mustard seeds, might also be capable of inducing goitrogenic effects in humans. However, no reliable studies exist regarding exposure of goitrogens due to the consumption of mustard and mustard products and higher risk of goiter or related pathologies.

Studies in human volunteers indicate that exposure of goitrogens (e.g., progoitrin) through the intake of realistic amounts of Brassica vegetables does not alter thyroid function. However, if consumed in considerable quantities it may contribute to development of goiter [74,75].

The absence of epidemiological evidence regarding goitrogenic effects after consumption of Brassica vegetables containing high amount of these toxic compounds, such as goitrin from Brussels sprouts, is partly due to the fact that cooking inactivates myrosinase responsible for activation of progoitrin to goitrin, thus reduces the biological availability of goitrogenic breakdown products to sub-clinical levels.

It needs to be emphasized, that mustard seeds or mustard condiment are generally not heat-treated and damage of the seeds might form high amounts of goitrogens whose exposition should still be considered and monitored, especially in iodine-deficient population groups or in subjects where thyroid hormone production is already impaired. Additionally, even if myrosinase activity is diminished, the ability of intestinal microorganisms to readily convert inactive precursors, such as progoitrin, into goitrogenic compounds, should also be considered.

#### 2.1.3. Genotoxic and Carcinogenic Effects

Certain glucosinolate breakdown products have been under observation as possible human carcinogens. Several of these compounds are electrophilically reactive, for example, due to the highly electrophilic central carbon in the isothiocyanate group (-N=C=S), and possibly undergo addition as well as substitution reactions with nucleophiles.

In the past, several isothiocyanates from Brassica vegetables including broccoli tested as freshly prepared juice were investigated using in vitro models and partially demonstrated dose-dependent genotoxic effects in bacterial and mammalian cells [76,77,78,79,80].

Musk et al. observed significant clastogenic activity (chromosome aberration and sister chromatid exchanges) in in vitro cell lines for phenyl isothiocyanate (PITC), phenethyl isothiocyanate (PEITC) and benzyl isothiocyanate (BITC), which is one of the major breakdown products of the glucosinolate sinalbin contained in white (*S. alba*) mustard seeds. No mutagenic effects were observed for allyl-isothiocyanate (AITC), known as the predominant hydrolysis product of sinigrin and the major flavour constituent of brown (*B. juncea*) and black mustard (*B. nigra*) seeds [81,82].

With regard to AITC, the EFSA Panel on Food Additives and Nutrient Sources added to Food (ANS) summarized in its scientific opinion on the safety of AITC used as a food preservative genotoxicity tests in in vitro and in vivo studies (summary of the studies in [83]). Positive as well as negative results have been observed in genotoxicity tests with AITC in bacterial and mammalian cells in vitro when no metabolic activation occurred. Mutagenic effects mostly occurred when cytotoxic concentrations of AITC were used. In the presence of metabolic activation, the results were generally negative, even in modified Ames tests with extended preincubation time suggesting that cytochrome P450 isoenzymes seem to be able to reduce markedly the occurrence of mutagenic derivatives. According to the EFSA Panel, reactive oxygen species might be involved in the genotoxic effects of AITC in vitro. On the other hand, no convincing evidence on genotoxicity was shown in in vivo studies performed on different genetic endpoints. Therefore, the Panel did not raise concern regarding genotoxic effects of AITC [83].

Moreover, AITC administered via gavage was not carcinogenic for B6C3F1 mice of either sex, however an increased incidence of transitional cell papillomas of the urinary bladder of F344/N male rats was reported [84]. Possible explanations for the carcinogenic effects of AITC at repeated high doses in male rats consider the accumulation of the corresponding mercapturic acid conjugate of AITC. The AITC conjugate *N*-acetyl-S-(*N*-allylthiocarbamoyl)-L-cysteine is the major urinary metabolite in humans and rats whereby in rats the AITC clearance is slower compared to humans. High concentrations of the N-*acetyl*-cysteine conjugate in the bladder might therefore be a direct irritant on the bladder epithelium or dissociate into free AITC likewise act as irritating substance resulting in regenerative hyperplasia and subsequent benign papillomas. Considering that no genotoxicity in vivo was observed, a threshold mechanism (high-dose response) of AITC underlying these effects in the urinary bladder was assumed [83].

Several aliphatic, aromatic and indole glucosinolates were also tested by Baasanjav-Geber et al. for their mutagenic potential using Ames test and two different *S. typhimurium* strains. Mutagenicity was observed only when myrosinase was added to the test compounds demonstrating the predominant role of the glucosinolate breakdown products with respect to the mutagenic potency. The indole glucosinolate neoglucobrassicin, mainly occurring in broccoli and kale, had the highest increases in the number of revertants in both strains. Sinalbin, which is the main glucosinolate in white mustard (*S. alba*) seeds and hydrolysed to 4-benzyl isothiocyanate (BITC), likewise had a high number of revertants. However, the mutagenic potency of sinalbin was much lower and similar to other glucosinolates tested [85].

In summary, high concentrations of several glucosinolates, especially indolic glucosinolates and their degradation products, isothiocyanates have been shown to be mutagenic in vitro and the results might give some cause for concern. However, clear genotoxic or carcinogenic effects in humans have not been reported [86,87]. In this context, it has to be emphasized that under in vivo test conditions isothiocyanates may be efficiently detoxified [26,76,78].

#### 2.1.4. Evaluation of Toxicological Effects

The characteristic hot and pungent flavour of mustard is based predominantly on isothiocyanates produced from the parent compounds sinigrin and sinalbin, though various types of other glucosinolate-derived products, such as goitrin, may also occur but in much smaller quantities. The described toxic and antinutritional effects of these biologically active breakdown products seem predominantly occur in animal species rather than in humans and with the administration of high doses.

In 2008, EFSA considered the Joint FAO/WHO Expert Committee on Food Additives (JECFA) evaluations of miscellaneous nitrogen-containing substances including allyl-isothiocyanate and benzyl-isothiocyanate. The panel agreed, based on a Maximised Survey-derived Daily Intake (MSDI) approach, with the JECFA conclusion that “No safety concern at estimated levels of intake as flavouring substances” is made, which also applied to allyl-isothiocyanate and benzyl-isothiocyanate, and that “the available data on genotoxicity and carcinogenicity do not preclude the evaluation of the flavouring substances through the Procedure” [88,89].

Except for AITC [83], no comprehensive toxicological evaluation and derivation of health-based guidance values of glucosinolates or their degradation products predominantly occurring in mustard seeds have been identified. In its opinion dealing with the safety of AITC for the proposed use as food additive, EFSA established an acceptable daily intake (ADI) of 20 µg/kg body weight. AITC concentrations in mustard seeds and products vary widely depending on mustard species and process conditions [10,57,83,90]. Assuming an average mustard consumption of 1 g/day containing 1000 mg AITC per kg mustard product, the intake of AITC would be 1 mg per day and would not exceed the ADI of 1.4 mg for an adult weighing 70 kg. However, if the body weight is lower or the AITC concentration is higher, up to 15,000 mg/kg mustard, as it is documented by EFSA [83], would result in exceedance of the ADI, e.g., for children or adolescents.

Numerous factors, such as the activity of myrosinase and specifier proteins as well as processing conditions (temperature, pH, acidic environment) may influence the conversion rate of glucosinolates to isothiocyanates and the formation of more or less, partially not yet known, toxic derivatives.

Early experimental data have indicated that isothiocyanates might be mutagenic and genotoxic in various assays, however evidence of carcinogenicity in experimental animals is inconclusive and human studies are limited. On the opposite, research has recently focused on the anti-carcinogenic properties of constituents of Brassica vegetables, especially of isothiocyanates. Anti-carcinogenic effects have been attributed to the inhibition of cytochrome P450 enzymes, and a concomitant induction of phase II detoxifying enzymes, thereby preventing the activation of pro-carcinogens and improving their conjugation and elimination [91,92,93].

Therefore, it is not surprising that glucosinolates and their hydrolysis products are already on the market as ingredients in dietary supplements and concentrated herbal preparations. However, most evidence concerning anti-carcinogenic effects and the mechanism of action has come from in vitro or animal studies.

Despite the huge number of epidemiological studies reporting on decreased cancer risk in humans along with a higher intake of Brassica vegetables, an increasing (therapeutic) use of high-dose, partially isolated glucosinolates (isothiocyanates) should be questioned when clear dose–effect relationships are not clinically proven and the tissue bioavailability is largely unknown. It is certainly possible that some glucosinolates have properties that are both carcinogenic and chemopreventive [94].

Furthermore, it has to be noticed that volatile mustard oils including AITC and goitrin are listed as undesirable substances in animal feed [61]. Pointing out the harmfulness of these compounds and the need of monitoring including exposure assessments when used by humans in concentrated forms is necessary because human studies focusing on adverse effects are still scarce. However, the intake of biologically active glucosinolate breakdown products from mustard seeds is comparatively low and could be considered to be tolerated when for example the ADI established for allyl isothiocyanates from whatever source is not exceeded.

### 2.2. Bisphenol F

Bisphenol F is an aromatic compound related to bisphenol A through its basic structure containing two phenol groups. The two aromatic rings of bisphenol F are linked through methylene. Generally, bisphenol F comprises several isomers such as 2,2′-, 2,4′- and 4,4′-dihydroxydiphenylmethane. Recently, bisphenol F was detected in several mustard products containing seeds of white mustard (*S. alba*) as well as in various plants of the orchid family (Orchidaceae) (*Coeloglossum viride* var. *bracteatum* (rhizome), *Galeola faberi* (rhizome), *Gastrodia elata* (rhizome), *Tropidia curculioides* (root)) and in the seeds of *Xanthium strumarium,* which are partly used as herbal remedies [95] (Table 3).

Bisphenol F is structurally similar to bisphenol A and belongs to one of the main substitutes replacing bisphenol A in the production of epoxy resins and polycarbonates used in the manufacture of adhesives, plastics, coatings and other applications [101]. Thus, the occurrence of bisphenol F is constantly increasing in products leading to consumer exposure from a broad number of sources.

In contrast to bisphenol A, bisphenol F in mustard and other plant species is not derived from synthetic materials, packaging or other sources of contamination, but is most likely formed as a breakdown product of glucosinolates [97].

#### Evaluation of Possible Health Risks

The occurrence of bisphenol F in mustard products is obviously due to the formation from a natural ingredient in the seeds during mustard production. Zoller et al. excludes the origin of bisphenol F from contamination through epoxy resin or other sources like food packaging. In support of this, the 4,4′-isomer was nearly exclusively detected in the samples. A contamination with bisphenol F would most likely lead to the occurrence of all isomers which are commercially used as a mixture of the three isomers, respectively [97].

It was observed that the highest levels of bisphenol F were detected in mild mustard samples compared to spicy ones. Additionally, bisphenol F was detected in mustard produced from seeds of *S. alba* but not from *B. juncea* or *B. nigra* or in mustard flour [97,99]. Therefore, based on differences in composition of the mustard species it is considered that bisphenol F is formed from the glucosinolate glucosinalbin, because it is predominantly found in the seeds of white mustard (*S. alba*) but not in *B. juncea* or *B. nigra*. This possibly can also explain the higher levels of bisphenol F in mild mustard types mainly contain *S. alba* which is milder in taste than other mustard species.

Recent findings from Zoller et al. verified the myrosinase-catalysed formation of 4-hydroxybenzyl isothiocyanate from 4-hydroxy benzyl glucosinolate (sinalbin). In the presence of water, the isothiocyanate is hydrolysed to the intermediate 4-hydroxybenzylalcohol. Subsequently, formation of a carbocation from the isothiocyanate by release of thiocyanate or from the 4-hydroxybenzyl alcohol by protonation and release of water is suggested. A further 4-hydroxybenzyl alcohol molecule could result in dimerization and formation of the 4,4′-isomer of bisphenol F [97]. Additionally, it was pointed out that 4,4′-bisphenol F could also be formed from 4-hydroxybenzylalcohol in the stomach due to its acidic conditions as it was also shown for other glucosinolate breakdown products [102].

After absorption bisphenol F is distributed throughout the body, also including the reproductive organs and the foetus by crossing the placental barrier. As glucuronide or sulfate conjugate, bisphenol F is rapidly excreted mainly via urine and to a lesser extent in faeces, however residues of bisphenol F were still detectable in tissues 96 h after a single dose was administered to rats [103].

So far, no comprehensive evaluation of possible health risks due to the exposure of bisphenol F in humans is available. Subchronic and chronic toxicity studies as well as studies on reproductive and developmental toxicity are still lacking. However, the structural similarity of bisphenol F to bisphenol A suggests comparable biological (adverse) effects and the potential to disrupt the human endocrine system [104,105,106,107,108].

In contrast to bisphenol A [104], no health-based guidance values for bisphenol F or legal regulations for foodstuff exist. However, some authorities evaluated the possible health risks to consumers from the natural occurrence of bisphenol F in mustard products. The Swiss authority FSVO (Federal Food Safety and Veterinary Office) carried out a risk assessment and evaluated the ratio between the lowest observed adverse effect level (LOAEL) and the dietary intake of bisphenol F from mustard products. In a very rudimentary approach, it was concluded that a daily intake of 0.67 mg of bisphenol F may be tolerable for a person of 60 kg body weight corresponding to a tolerable daily intake of 11 µg/kg body weight/day. The calculation was based on a LOAEL (Lowest Observed Adverse Effect Level) of 20 mg/kg body weight/day which was the lowest dose tested in a repeated 28-day toxicity study in rats leading to reduced body weight and several changes of blood parameters. Including several extrapolation factors (from LOAEL to NOAEL (No Observed Adverse Effect Level); from subacute to chronic toxicity) and an uncertainty factor of 100, it was assumed that consumption of 80 g mustard with the highest detected level of bisphenol F (8.4 mg/kg) would pass the margin of safety of 1800 which was postulated by the authority [98].

The German Federal Institute for Risk Assessment (BfR) used for its assessment the tolerable daily intake (TDI) of bisphenol A of 4 µg/kg body weight/day which was established by the European Food Safety Authority (EFSA) in 2015 [104] and is currently being re-evaluated [109]. The German authority concluded that even in the case of consuming high amount of mustard (4 g/day) with a maximum bisphenol F content of 6 mg/kg, the daily mean intake of bisphenol F would not exceed the assumed TDI of 4 µg/kg body weight/day. In the case of “normal consumers”, the estimated intake quantity is 100 times lower [110]. Therefore, adverse health effects due to the intake of bisphenol F from mustard are not to be expected, although further toxicological studies are needed for a final evaluation. It should be underlined that bisphenol A substitutes appear to have similar metabolism, potencies and action and may pose similar potential health risks as bisphenol A. Thus, consumption of mustard may be an important source of bisphenol F and continues monitoring of this compound and its relevant sources, either natural or technical-induced, and thorough investigations on its health effects in humans remain important [100,111,112].

### 2.3. Erucic Acid

Mustard seeds are regarded as an important source of edible oil, which are used due to its nutty and pungent flavour and its high smoke point (250 °C), especially in Eastern and North-Western India [113]. Oil from Brassica plants differ from other vegetable oils mainly due to their significant proportion of long-chain monoenoic fatty acids, eicosenoic and erucic acids. Wendlinger et al. detected up to 19 fatty acids in mustard oils, of which oleic acid (18:1n-9), linoleic acid (18:2n-6), alpha-linolenic acid (18:3n-3), eicosanoic acid (20:1n-9) and erucic acid (22:1n-9) belong to the major ones [114]. Erucic acid is a typical example of a very-long chain mono-unsaturated fatty acid (VLCMFA) with 22 carbon atoms and one double bond between C13 and C14, also referred to as docosenoic acid (C22:1). Further, it belongs to the predominant fatty acid in seed oils from the three commercial cultivated mustard species *B. nigra, B. juncea* and *S. alba* containing levels over 30% erucic acid of total fatty acids (Table 4).

The fatty acid profile and erucic acid content of mustard seeds depend on several factors such as breeding techniques, total oil content, climatic conditions as well as morphological and physiological determinants [116]. Recently, breeding programs has successfully produced canola quality (low, zero erucic acid content) of *B. juncea* and low erucic acid content genotypes are cultivated in few countries [126]. Nevertheless, mustard seeds and mustard oils are one of the food products with the highest erucic acid content that can be found on the market. Based on the low consumption rate of mustard oil in the European Union, mustard seeds and mustard condiment or table mustard remain here the major sources regarding erucic acid intake [127].

#### 2.3.1. Toxicological Effects

Like other long-chain fatty acids, erucic acid is transported to the tissues either bound to serum albumin or in esterified form incorporated in lipoproteins. The heart and skeletal muscles primarily utilize fatty acids as energy-providing substrates mainly through mitochondrial ß-oxidation. However, the capacity for mitochondrial ß-oxidation is reduced for long chain fatty acids (>18 carbon atoms). In this case, erucic acid is likewise sparsely oxidised by the mitochondrial β–oxidation system, probably due to the poor utilization of erucoyl-CoA as substrate by the mitochondrial acyl-CoA dehydrogenase. Furthermore, erucic acid also appears to inhibit the overall rate of fatty acid oxidation, by the mitochondria. It is worth noting, that both the capacity for ß-oxidation and inhibition of the tricarboxylic acid cycle differs between species. Rats compared to pigs seem to have a lower capacity for ß-oxidation and higher ability to inhibit oxidation of tricarboxylic acid-cycle intermediates [128,129].

Therefore, high exposure of erucic acid leads to accumulation of triacylglycerols in heart and other tissues. It has been shown that the lipidosis is reversible and transient during prolonged exposure, probably due to the increased peroxisomal chain shortening mediated by peroxisome proliferator-activated receptors. However, myocardial lipidosis is the most common and sensitive effect associated with short-term, and to a lesser extent, sub-chronic exposure of erucic acid in all animal species examined. With respect to liver tissue, the presence of erucic acid appears to induce the peroxisomal β–oxidation system, resulting in no accumulation of erucic acid and in reduced inhibition of mitochondrial ß-oxidation. Consequently, the liver is able to export erucic acid as very low-density lipoprotein (VLDL) [130,131,132,133].

Given what is known about the metabolism of erucic acid, it seems reasonable to expect that humans would also be susceptible to myocardial lipidosis following exposure to high levels of erucic acid. Clouet et al. observed a very low capacity in human heart mitochondria for the direct utilization of erucic acid as a substrate for energy requirements. Furthermore, activation of fatty acids due to the transfer of acyl groups from Coenzyme A (CoA) to carnitine, which belongs to the preliminary step of their beta-oxidation, was also reduced with high erucic acid exposure [134].

Imamura et al. showed that higher levels of docosenoic (22:1) and nervonic (24:1) acids in plasma phospholipids from diverse dietary sources, were associated with higher incidence of congestive heart failure in two independent cohorts, assuming possible cardiac toxicity of long-chain monounsaturated fatty acids [135]. In contrast, Matsumoto et al. described a lower incidence of coronary heart disease with increased levels of erucic acid in erythrocytes [136].

According to EFSA, treatment with Lorenzo’s oil, which is a mixture of omega-9 fatty acids (oleic and erucic) to normalize the accumulation of very long chain fatty acids in adrenal and cerebral tissues in patients with adrenoleukodystrophy, led to undesirable effects on the hematopoietic system at doses of 100 mg/kg body weight per day [127].

So far, no reliable information is available regarding the development of myocardial lipidosis after high intake of erucic acid in humans. Epidemiological studies show no clear association between cardiac disease in humans and a diet high in erucic acid. One has to notice, that erucic acid metabolism is relatively complex in respect of differences in organ dependent metabolism of long chain fatty acids including lipid incorporation, chain shortening and elongation.

The EFSA Panel on Contaminants in the Food Chain (CONTAM) noted, that erucic acid-induced myocardial lipidosis observed in several animal species may also be relevant in humans, especially with the background that myocardial lipidosis is associated with cardiac insufficiency [127,137]. Therefore, oils, including high erucic acid content, are considered undesirable for human consumption. In 2016, EFSA conducted a comprehensive toxicological review and risk assessment of erucic acid in food and feed and established a tolerable daily intake (TDI) for erucic acid of 7 mg/kg body weight. The TDI is based on the observed development of myocardial lipidosis in juvenile rats which were treated for 7 days with 1 g erucic acid/kg body weight, and in neonatal piglets that received 1.1 g/kg erucic acid for 14 days [131,132]. Higher doses of erucic acid resulted in adverse effects on liver, kidney, skeletal muscles and caused changes in body and testis weight. Moreover, higher erucic acid intake was accompanied by mitochondrial damage and disorganisation of myofibrils as well as higher incidence of myocardial necrosis and fibrosis. No conclusion on genotoxicity and carcinogenicity could be made by EFSA due to limited data available. A single generation reproductive study was performed in rats and guinea pigs where doses of erucic acid up to 7500 mg/kg body weight/day were not associated with any adverse reproductive or developmental effects [127].

#### 2.3.2. Evaluation of Toxicological Effects

Since it was reported that consumption of oils rich in erucic acid are accompanied with the onset of myocardial lipidosis and heart lesions in a number of species, erucic acid content in edible oils consumed by humans was restricted to certain levels by various regulatory agencies.

The Australia New Zealand Food Standards Code (FSANZ) has considered erucic acid as natural toxicant and set a maximum level of 20 g/kg (2%) in edible oils [138]. This also applies for the European Union. Commission Regulation (EU) 2019/1870 sets a maximum limit of 20 g/kg (2%) for erucic acid in vegetable oils and fats placed on the market for the final consumer or for use as an ingredient in food, whereas the maximum permitted level for mustard oil is 50 g/kg (5%).

In its scientific opinion, EFSA concludes that the 95th percentile of dietary exposure levels of erucic acid is especially high in infants and other children. For highly exposed children, this may pose an elevated health risk [127]. It has to be noted, that stricter levels apply to infant formula and follow-on formula (Commission Delegated Regulation (EU) 2019/828). The use of mustard (or the oil) as ingredient in baby food is not usual, however due to its numerous functional properties the utilization of small amounts cannot be excluded.

For mustard as condiment a maximum level of 35 g/kg (3.5%) was established, however “with acceptance from the competent authority, the maximum level does not apply to mustard oil locally produced and consumed”. In the European Union level, no maximum permitted level for erucic acid is set for mustard seeds or powder, which may contribute to a high exposure of the undesirable substance. In contrast to Asiatic countries, use of mustard oil in Europe’s cuisines is rare and mustard is mainly consumed in the form of prepared (table) mustard.

The U.S. Food and Drug Administration (FDA) banned mustard oil in pure form by publishing an Import Alert specifically stating that “Expressed mustard oil is not permitted for use as a vegetable oil. It may contain 20 to 40% erucic acid, which has been shown to cause nutritional deficiencies and cardiac lesions in test animals” [139].

According to the literature search on erucic acid content in mustard seeds and products derived from (Table 4), the findings indicate that both seeds and oils represent relevant sources for human intake. Wendlinger et al. showed in 2014 that most of the mustard oil samples analysed were well above the European Union limit of 5% erucic acid [114]. Recently, the same group from Germany addresses the intake of erucic acid from different sources including table mustard. According to the authors, teenagers and adults may exceed the tolerable daily intake of 7 mg/kg body weight/day by consuming one serving of mustard (10 g) depending on the contribution of erucic acid to the total fatty acids (>40%) of the seeds and the lipid content of the prepared product (>10%) [117].

It seems obvious that mustard consumption may contribute considerably to the intake of erucic acid. Especially in India and Pakistan, the use of mustard oil as cooking oil is widely distributed and still contain high amount of erucic acid. Several experts from India arguing that the health risks associated with mustard oil, also called the “Olive oil of India” were not observed in the Indian population, probably due to the high alpha-linolenic acid content which might compensate for the erucic acid [140]. Nevertheless, further development of mustard breeds low in erucic acid and a careful choice of the mustard cultivar used in food products will be in favour of consumer protection.

### 2.4. Allergens

Mustard seeds have a relatively high protein content, up to 36% depending on the mustard species. The mustard seed storage proteins 2S albumin, referred to as napin, are highly abundant in mustard seeds, and have been identified as major mustard allergens with significantly higher response in allergy tests compared to other storage proteins [141,142].

Allergenic 2S albumins have been characterized from white mustard (*S. alba*) [143,144] and brown mustard (*B. juncea*) [145]. According to the WHO/IUIS Allergen Nomenclature Subcommittee [146] the proteins are termed as Bra j 1 and Sin a 1 which show a close sequence homology [147] which increases the probability that people allergic to one mustard species are also sensitive for the other one [145]. For black mustard (*B. nigra*), one of the progenitor species of *B. juncea*, no information on allergens have been provided by the WHO/IUIS Allergen Nomenclature Subcommittee, however it can be assumed that seeds likewise contain allergenic 2 s albumins [148].

Beside the seed storage protein from the 2S albumin, three more allergens have been identified in *S. alba* including Sin a 2 which belongs to the seed storage 11S globulin and known as cruciferin; Sin a 3 corresponding to a non-specific lipid transfer protein, and Sin a 4, named as profilin (Table 5) [149].

Recently, Hocine et al. assessed protein profiles and immunoglobulin E (IgE)-binding patterns of selected mustard varieti es (*S. alba* and *B. juncea*). In addition to proven allergens, the authors identified other new IgE-binding protein bands from *S. alba* and *B. juncea* varieties [150].

At present, no effective preventive treatments exist for mustard allergy. The allergenic proteins are highly resistant to heat treatment. Thermal denaturation for napin and cruciferin occurs upon 80 °C depending on the pH-value [151]. Moreover, mustard allergens are poorly digestible. Sin a 1 was shown to be resistant to digestion by trypsin and other proteolytic enzymes [141,152]. Therefore, the allergenic potential cannot be effectively reduced during mustard processing or preparation of table mustard.

Previous studies suggest that allyl-isothiocyanate (AITC), which derives from sinigrin mainly contained in *B. nigra* and *B. juncea* could have the potential to cause allergic contact dermatitis in humans [153,154]. EFSA evaluated the safety of AITC when used as food preservative and concluded that AITC may cause contact hypersensitivity which is an immunologically mediated adverse reaction but mechanistically different from food allergy, although it might be extremely unlikely that AITC acts as a direct food allergen [83]. In the scientific opinion on the evaluation of allergenic foods and food ingredients for labelling purposes, EFSA additionally pointed out that mustard may contain a number of further irritants triggering non-immune mediated reactions mimicking allergic reactions [155].

#### 2.4.1. Clinical Indication

Food allergy can be IgE-mediated or non-IgE-mediated. IgE-mediated reactions have a rapid onset, affecting skin, respiratory and gastrointestinal tract, and in some cases can lead to systemic anaphylaxis, whereas non-IgE-mediated food allergy usually is delayed and affects mainly the skin and the gastrointestinal tract. The allergic symptoms after consuming mustard or mustard-containing products are comparable to other IgE-mediated food allergies. This includes allergic or atopic dermatitis resulting in skin reactions such as rashes or hives, urticaria, swelling of lips and tongue, facial flushing and oedema, chest tightness and respiratory problems, rhinitis, asthma, nausea and vomiting, dizziness and anaphylactic shock. Mustard allergy symptoms were also observed in children under the age of 3 years indicating a primary sensitization to mustard in at least some food allergies [148,156,157,158].

It is suggested that approximately 50% of patients allergic to mustard are also sensitized to mugwort pollen (*Artemisia vulgaris*) and several vegetable foods, mainly from the Rosaceae family, but also from tree nuts, peanuts and legumes [159]. Vereda et al. showed that 21 out of 34 subjects sensitized to mustard had cross-reactivity with fruits from the Rosaceae family (peach, apple, pear, apricot, plum, cherries and strawberries, excluding almond) and 20 suffered from allergy to one or more nuts. Moreover, allergy to mugwort was especially reported [160]. Sin a 1 was the most prevalent allergen and highly correlated with specific IgE levels. Therefore, Sin a 1 is considered the major allergen of white mustard *(S. alba*) and the most suitable marker for a precise diagnostic screening of mustard sensitization.

Figueroa et al. reported that all of the 38 patients with mustard IgE-mediated hypersensitivity showed associated sensitization to other members of Brassicaceae family, and cross-reactivity among them was confirmed. However only 40% of these had symptomatic reactions, mainly to cabbage, cauliflower and broccoli [161].

The reported cross-reactivities with pollens or with members of the Brassicaceae family may influence the positivity of specific IgE and skin-prick tests. This may result in overestimated prevalence of sensitization to mustard, and may possibly influence the occurrence of oral allergy syndrome-like symptoms elicited by mustard. Another factor contributing to a possible overestimation of mustard sensitization and allergy may be the presence of irritants in mustard preparations or mustard containing products. For instance, the neuropeptide-active agent capsaicin may affect the release of substance P causing a non-IgE mediated mast cell degranulation [156,162]. Therefore, irritants such as capsaicin or isothiocyanates may trigger non-immune reactions mimicking allergic reactions which may lead to false positive allergy-like reactions. For example, Morisset et al. have shown that only 23.3% of patients with positive skin prick tests are truly allergic to mustard based on positive oral food challenges [148].

#### 2.4.2. Evaluation of the Allergic Potential

Mustard is one of the priority food allergens declared under several international food allergen labelling regulations, for instance in the European Union or Canada but not in the U.S. [163]. According to Regulation (EU) No 1169/2011 mustard belongs to one of the 14 major allergens that shall be indicated in the list of ingredients. The inclusion of mustard was based on the view that mustard allergy is a serious health problem in certain countries, although limited data are available according to the number of people affected. Mustard allergy is recognized as one of the most frequent spice allergies with a credible cause–effect relationship confirmed by single and double-blind placebo-controlled food challenges (DBPCFC). Several clinical studies and case reports documented severe systemic reactions, including anaphylactic reaction immediately after the ingestion of mustard, even after small quantities [164,165,166]. Only a small number of DBPCFC or oral food challenges (OFC) studies exist in the literature, which possibly can be justified by technical difficulties of masking the strong taste of mustard limiting the attempts to perform these studies. Previously, it was suggested that mustard allergy accounts for about 1% of food allergies in children and up to 7% of total food allergies based on estimated prevalence in France [152,155,167].

The amount of mustard required to elicit an allergic reaction may be very small. Estimations of the eliciting dose range from a mean cumulative reactive dose of mustard sauce of 891.4 ± 855.2 mg, equivalent to 124.8 ± 119.7 mg of mustard, published by Figueroa et al. to 40 mg of mustard seasoning (roughly equivalent to 0.8 mg of protein) reported by Morisset et al. [148,161]. In 36 children with a positive mustard skin-prick test, the cumulative reactive dose by open challenge or double-blind placebo controlled food challenge (SBPCFC) varied from 1 to 936 mg [156]. Most of the case reports indicate that allergic reactions come from mustard sauce or mustard hidden in other products, such as mayonnaise, dips, ketchup sauces, where mustard is present in small or trace amounts [164].

Using data from clinical studies and mathematical calculations the VITAL Scientific Expert Panel (VSEP) recently published an update on reference doses including mustard allergens. For mustard, 0.05 mg protein has been derived as a reference dose for ‘ED01’, or 0.4 mg protein for ‘ED05’ corresponding to a dose where 99% or 95% of people affected by a mustard allergy are protected from developing objectively measurable allergic reactions [168]. This is in line with EFSA’s conclusion that 1 mg mustard protein may trigger allergic reactions in patients allergic to mustard [155].

Mustard is widely used in foods due to its sensory attributes, its high protein content and its numerous functional properties. Therefore, foods formulated with mustard are expected to rise in future and the risk of masked allergens in modern food products is increasing. In regions with high consumption, such as France, mustard might be among the most important food allergen for children. Hence, it deserves special attention, particularly as a potent “hidden allergen” provoking unexpected allergic reactions. The seriousness of the allergic reactions argues for informative labelling in countries where mustard is not considered as food allergen, such as the U.S.

## 3. Conclusions

Mustard seeds are widely used in foods due to its sensory attributes, nutritional values, and its numerous functional properties. The intake of mustard products and foods formulated with mustard is expected to rise in future and may contribute considerably to the exposure to several biologically active compounds that were objects of this work. Mustard products and their seeds are consumed in very small quantities primarily explainable by the characteristic hot and pungent flavour. Adverse effects of bioactive compounds such as erucic acid or glucosinolates breakdown products have been mainly observed in in vitro or in vivo animal studies rather than in humans and especially when the compounds were administered in concentrated or isolated form, and given in high doses. When consumed in average amounts typically found in a normal diet, it can be expected, that the intake of mustard seeds or products made thereof do not pose a direct health risk, except for consumers allergic to mustard proteins. However, it cannot be excluded that high intake levels of mustard seeds or products made thereof, such as mustard seed oil, cause health problems. Nowadays, research is basically focusing on the potential beneficial effects of the multifunctional mustard plant and its ingredients, however reliable data on potentially toxic compounds and possible health risks should likewise be considered.

## Figures and Tables

**Table 1:** Distribution of glucosinolates among mustard plant species.

Mustard Species	Common Names	Glucosinolates Identified	Systematic Name
		Trivial Name	(a-Glycone = R Side Chain)
*S. alba*	white, yellow mustard	Gluconapin ^a^	3-Butenyl
		Progoitrin ^a^	2R-2-Hydroxy-3-butenyl
		Glucobrassicanapin ^a^	Pent-4-enyl
	
		# (Gluco-)Sinalbin ^b^	4-Hydroxybenzyl
		Glucotropaeolin ^b^	Benzyl
		Gluconasturtiin ^b^	2-Phenylethyl
	
		Glucoerucin ^c^	4-Methylthiobutyl
		Glucoibe(rve)rin ^c^	3-Methylthiopropyl
		Glucoiberin ^c^	3-Methylsulphinylpropyl
	
		2-Methylpropyl Isobutyl ^d^	
	
		Glucobrassicin ^e^	3-Indolylmethyl
		Neoglucobrassicin ^e^	N-Methoxy-3-indolylmethyl
*B. nigra*	wlack, shortpod mustard, moutarde noire	# (Gluco-)Sinigrin ^a^	2-Propenyl
*B. juncea*	brown, indian, asiatic, chinese, sarepta mustard	# (Gluco-)Sinigrin ^a^	2-Propenyl
		# Gluconapin ^a^	3-Butenyl
		Progoitrin ^a^	2*R*-2-Hydroxy-3-butenyl
		Epiprogoitrin ^a^	2*S*-2-Hydroxy-3-butenyl
	
		Glucosinalbin ^b^	4-Hydroxybenzyl

# predominant glucosinolates in mustard seeds; ^a^ aliphatic olefin, ^b^ aromatic aryl, ^c^ S-containing, ^d^ aliphatic branched chain, ^e^ aromatic indol.

**Table 2:** Glucosinolate content in mustard species.

Mustard Species	Total Content of Glucosinolates	Content of Predominant Glucosinolates	Reference
*S. alba*		Sinalbin (seed): 250 µmol/g sinalbin	[23]
		Sinalbin (seed): 165 ± 3 µmol/g Sinigrin (seed): 155 ± 4 μmol/g	[41]
	Seed: 45.4–61.9 g/kg FW	Sinalbin (seed): 16.6–46.2 g/kg FW Progoitrin (seed): 0.8–1.3 g/kg FW Glucoibervirin/Glucobrassicin/4-hydroxglucobrassicin (seed): <0.2 g/kg FW	[42]
*B. nigra*	Seed: 207.2–229.0 µmol/g FW	Sinigrin (seed): 202–226.7 µmol/g FW	[43]
	Seed: 26–62.4 g/kg FW	Sinigrin (seed): 24.5–61.2 g/kg FW Gluconapin/Progoitrin/Glucoibervirin (seed): <0.3 g/kg FW Sinalbin/4-hydroxglucobrassicin (seed): <0.9 g/kg FW	[42]
*B. juncea*	Intact seed: 15.7–127.6 µmol/g DW Leaf: 4.3–129.9 µmol/g DW	Gluconapin: (leaf): 0.6–70.1 µmol/g DW (seed): 2.3–90.8 µmol/g Sinalbin (leaf): 0.1–64.9 µmol/g DW (seed): 0.2–35.2 µmol/g Epiprogoitrin (leaf): 0.7–16.4 µmol/g DW	[44]
	Seed: 94.4–169.2 µmol/g FW	Sinigrin (seed): 43.79–145.5 µmol/g FW Gluconapin (seed): 0–110.58 µmol/g FW	[43]
	Seed: 68–153 μmol/g FW	Sinigrin (seed): 0–134.2 μmol/g FW Gluconapin: 68–153 µmol/g FW	[45]
	Defatted seed meal: 58.83–132.26 μmol/g		[46]
	Oil free seed meal: 123.8–152.1 µmol/g		[47]
		Sinigrin (seed): 155 ± 4 µmol/g	[41]
	Seed: 18.1–61.8 g/kg FW	Sinigrin (seed): 16.6–46.2 g/kg FW Gluconapin/Progoitrin/Glucoibervirin/Glucoerucin/ Sinalbin/Gluconasturiin (seed): <1 g/kg FW 4-hydroxyglucobrassicin (seed): 0.6–1.6 g/kg FW	[42]

DW dry weight, FW fresh weight.

**Table 3:** Occurrence of bisphenol F in foods related to mustard.

Food Items	Bisphenol F Content (in mg/kg)	Reference
Condiments	2.4	[96]
Commercial table mustard (n = 61)	mean 1.84 (max 8.4)	[97,98]
thereof mild mustards (n = 19)	mean 3.2	
Commercial table mustard		[99]
Medium hot mustard (n = 39)	<5 (n = 37); >9 (n = 2)	
Sweet mustard (n = 7)	<3	
Hot mustard (n = 10)	<3	
Quince mustard (n = 1)	<1	
Self-made mustard products		
Mustard flour	n.d.	
Table mustard from *B. nigra*	n.d.	
Table mustard from *B. juncea*	n.d.	
Table mustard from *S. alba*	0.18–0.94	
Mustard dressing	1.13	[100]

n.d. not detected.

**Table 4:** Erucic acid concentration in mustard products.

Type		Origin	Amount of Erucic Acid (of Total FA)	Reference
Mustard seed	*B. juncea* mustard (54 varieties)	India	35.7–51.4%	[113]
	*B. juncea* varieties	Bangladesh	43.7–48.6%	[115]
	*B. juncea* (51 collections)	India	22.5–53.4%	[116]
	*B. nigra* (3 collections)		35.9–40.1%	
	*S. alba* (4 collections)		39.1–47.2%	
	*B. nigra* (n = 5)	Germany	20–40%	[117]
	*B. juncea* (n = 5)			
	*S. alba* (n = 5)			
	*B. juncea* (n = 12)	India	39.4–51.8%	[46]
	*B. juncea* (n = 3)		0.9–1.6%	
	*B. nigra* (n = 5)	Egypt	33.3–45/32–42.9 %^a^	[118]
	*B. juncea* (n = 5)		21.9–40/24.2–42.7 %^a^	
Mustard seed oil	Mustard oil (n = 3)	Australia	43.8–45.8%	[114]
	Mustard oil (n = 6)	Germany	0.3–50.8%	
	*S. alba* mustard oil	Egypt	37.90%	[90]
	*B. juncea* mustard oil		23.90%	
	Mustard oil	India		[119]
	Variety 1 (n = 3)		0.9–4.7%	
	Variety 2 (n = 2)		14.7–34.1%	
	Mustard oil	Bangladesh	41.80%	[120]
	Mustard oil (Erucic free)		20.10%	
	Mustard oil (n = 59)	India	48.5–54.02%	[121]
	Mustard oil	Turkey	11.40%	[122]
	Mustard oil	Malaysia	18.50%	[123]
	*S. alba* mustard oil	Poland		[124]
	Cultivar 1		22.20%	
	Cultivar 2 (Erucic free)		3.80%	
Other mustard products	Mustard samples (n = 15)	Germany	14–33%	[114]
	Mustard sauces (n = 5)		<5%	
	Mustard condiment	Germany	1.6 g/100 g condiment ^b^	[125]
	Mustard seeds		11.5 g/100 g seeds ^c^	
	Mustard sauces	Malaysia	33.10%	[123]
	Mustard sauces		32.20%	
	Mustard powder		25.50%	

^a^ 2 crop years, ^b^ total fat content 4%, ^c^ total fat content 28.8%.

**Table 5:** Mustard allergens.

Mustard Species	List of Allergens	Type	Source ^a^
*S. alba*	Sin a 1	2S albumin	http://www.allergen.org
	Sin a 2	11S globulin (legumin-like) seed storage protein	http://www.allergen.org
	Sin a 3	Non-specific lipid transfer protein type 1 (ns-LTP)	http://www.allergen.org
	Sin a 4	Profilin	http://www.allergen.org
*B. nigra*	Bra j 1	2S albumin seed storage protein	http://www.uniprot.org *
*B. juncea*	Bra j 1	2S albumin seed storage protein	http://www.allergen.org

^a^ accessed on 8 June 2021, * not listed according to the WHO/IUIS Allergen Nomenclature Subcommittee.

## Data Availability

Not applicable.
